# Supplementary material for: Neurosyphilis: Still prevalent and overlooked in an at risk population
Source: PLoS One. 2020 Oct 7;15(10):e0238617. doi: 10.1371/journal.pone.0238617 (PMC7540903; doi:10.1371/journal.pone.0238617)
Supplement: S1 File — (DOCX) [file pone.0238617.s001.docx]

Table 1: CDC 2014 Criteria

Table 2: CDC Criteria 2018

Table 3: Cases of syphilis in the Indigenous and non-Indigenous populations, categorized based on onset. The majority of cases prior to 2013 were late latent NS with declining number. The sharp rise in numbers in the Indigenous population after 2013 are due to new cases. In 2015 there was a change in case definition to allow high initial RPRs to be categorised as “probable” in the  “< 2 years” category – rather than “> 2 years or of unknown duration”, the higher proportion of cases in the “< 2 year” category from 2015 onwards was due to the change in definition.

|  | Onset < 2 years | Onset < 2 years | Onset < 2 years | Onset > 2 years or of unknown duration | Onset > 2 years or of unknown duration   \|  \| \| --- \| \| | Onset > 2 years or of unknown duration |
| --- | --- | --- | --- | --- | --- | --- | --- |
|  |  |  |  |  |  |  |
|  | **Indigenous** | **Non-Indigenous** | **Unknown** | **Indigenous** | **Non-Indigenous** | **Unknown** |
| 2007 | 16 | 10 | 3 | 106 | 9 | 1 |
| 2008 | 23 | 15 |  | 107 | 12 |  |
| 2009 | 22 | 1 |  | 50 | 18 |  |
| 2010 | 16 | 3 |  | 39 | 21 | 3 |
| 2011 | 12 | 2 |  | 15 | 22 | 3 |
| 2012 | 5 | 1 |  | 12 | 42 | 2 |
| 2013 | 1 | 10 |  | 10 | 58 | 1 |
| 2014 | 22 | 11 |  | 21 | 19 |  |
| 2015 | 98 | 20 |  | 36 | 13 | 1 |
| 2016 | 154 | 22 |  | 24 | 21 | 2 |
