# Supplementary material for: Neurosyphilis: Still prevalent and overlooked in an at risk population
Source: PLoS One. 2020 Oct 7;15(10):e0238617. doi: 10.1371/journal.pone.0238617 (PMC7540903; doi:10.1371/journal.pone.0238617)
Supplement: Attachment — Submitted filename: Reply to Reviewers_final.docx [file pone.0238617.s002.docx]

The rate of lumbar puncture was 2.4% - do you know how this compares with other countries ?

**Thank you for this excellent suggestion. Unfortunately, it is not clear after reviewing the literature what LP rates were in other countries. As we one of our methods of screening for NS patients was by reviewing all LP performed in the region we were able to arrive at those figures. Other studies have either determined NS through chart reviews, referrals to tertiary centers or reporting to their local CDC. These cases have already met criteria for NS. It is unclear from these studies how many patients had an LP for a query of NS but did not met criteria, and therefore not diagnosed with NS.**

Is there a take home message for practicing clinicians, given the low rate of appropriate treatment ?

**We have added a line to the conclusion to try address this. Line 386**

Could you add a line or two telling us what you think the limitations are of your study, please ?

**We have added a paragraph explaining the limitations of the study, including the difficulties in making a definitive diagnosis of NS and our reliance on mostly retrospective data. Line 373**

The authors estimate incidence rates in their population. While they comment on the risk of overestimate in their discussion due to the inclusion of probable cases, they should also comment on the possibility that these are underestimates (at least using the diagnostic criteria that have been employed). Neurosyphilis is often asymptomatic or not considered in the differential diagnosis. Unless the entire population was screened by anti-treponemal antibody, one really does not know the true prevalence of infection.

**We agree that this is an important point. We have now raised the issues in the discussion along with the comments regarding asymptomatic cases. This is on line 280 of the discussion.**

The authors state that the incidence of syphilis declined significantly following the introduction of penicillin. While this is true, contact tracing also played a significant role in the decline in incidence in the U.S. as case rates began falling long before the widespread institution of penicillin.

**Thank you for this addition and something we have now mentioned with appropriate referencing on line 90**

The increased incidence in the indigenous population, presumably individuals in a lower socioeconomic group relative to the white population, parallels the experience in the U.S. where rates of syphilis is substantially higher in the African-American and Hispanic populations.

**Yes, this is indeed true, the Australian Indigenous population are individuals who have a considerably lower socioeconomic background compared to Whites, with poorer health outcomes. We have added an additional line regarding this in our discussion (line 313).**

Two of their patients were HIV-infected. Can they state what percentage of their patients were men who have sex with men among both the indigenous and non-indigenous populations in their study?

**Unfortunately, we do not have that data as it was not consistently or clearly documented in the medical records. It was not available to us from the data provided from the CDC either.**

Were there not any asymptomatic neurosyphilis cases? If not, why not? Also, can the authors identify whether the neurosyphilis manifestations were early or late manifestations of the infection. Whereas dementia, tabes, and psychosis are typically late manifestations (tertiary syphilis), meningitis, seizures and uveitis are often seen with secondary syphilis, particularly, in the HIV infected population.

**There were no cases of asymptomatic NS, which was likely due to our methodology of screening cases based on LPs performed, all of who had neurological symptoms. It also likely relates to the practice of not consistently testing for NS with an LP in patients with syphilis that fail to have a four-fold decrease in RPR despite treatment. There is probably a much higher prevalence of asymptomatic cases that we have failed to detect. This has been added to the discussion (line 282) as well as the results section (line 222). We have categorized cases into early and late manifestations and added it to the results section (line 220), 79% of cases were late manifestations.**

How comfortable are the authors in attributing the neurological manifestations to syphilis? After all, “a man can have as many diseases as he damn well pleases.” If the clinicians caring for these patients felt comfortable that there were better explanations for their neurological manifestations might that not explain why those with “probable neurosyphilis” were not treated?

**This is an excellent point that was raised amongst our group. We have added our thoughts regarding this in the discussion (line 340), specifically around the issue of dementia, which is a common condition in the general population. If the clinician felt that the cause of dementia was due to a more common or likely cause then they may not have treated a diagnosis of probable NS. As we do not have in-depth cognitive testing results on our patient cohort, it is difficult for us to say that there was not another dementing syndrome. Further prospective studies are planned to address this.**

“the test carries a high sensitivity and low sensitivity” (not “now”) line 129

**Thank you, correction made**

How do the authors explain the apparent profound decline in syphilis in the indigenous population between 2007 and 2015 with a nadir in 2013, a year that the incidence in the non-indigenous increased?

**The vast majority of cases between 2007-2015 were late latent cases of syphilis in both the Indigenous and non-Indigenous population. These cases were on the decline in the indigenous population, which we believe was in part due to the work by the CDC, though this is difficult to say in retrospect. The small rise in 2013 in the non-indigenous population is in late latent cases as well, though we are not sure why there was a rise. The sharp increase in cases from 2013 onwards are all new cases of syphilis in the Indigenous population, with late latent cases remaining stable in both groups. We have added a table with a breakdown of the raw numbers as a supplementary table for additional clarity.**

How does the application of the 2018 CDC criteria change the numbers with respect to the percentage of probably neurosyphilis in which there was a failure to treat?

**Thank you for this suggestion as it was not something we had thought to look at. In fact with the 2018 CDC criteria only 3/10 cases received IV BP and 3 cases received IM benzathine. This is less than with the 2014 criteria. We have added these findings to the abstract and discussion section (line 74 & 369).**
